# Reference equations for the six-minute walking distance in obese Chinese subjects more than 40 years old

**DOI:** 10.1007/s40519-022-01404-8

**Published:** 2022-04-22

**Authors:** Jia Zhang, Yingying Zou, Zibin Wang, Xiaoshu Chen, Jingye Pan, Haizhu Yu, Cong Lin, He Zou

**Affiliations:** 1 Department of Inspection Medical, Wenzhou People’s Hospital, The Wenzhou Third Clinical Institute Affiliated with Wenzhou Medical University, Wenzhou, Zhejiang China; 2 Digestive System Department, The Third Affiliated Hospital of Qiqihar Medical College, Qiqihar, Heilongjiang China; 3 Obstetrics Department, Wenzhou People’s Hospital, The Wenzhou Third Clinical Institute Affiliated with Wenzhou Medical University, Wenzhou, Zhejiang China; 4 Department of Cardiovascular Medicine, Wenzhou People’s Hospital, The Wenzhou Third Clinical Institute Affiliated with Wenzhou Medical University, Wenzhou, Zhejiang China; 5 Department of General and Intensive Care Medical, The First Affiliated Hospital of Wenzhou Medical University, Wenzhou, Zhejiang China; 6 Department of General Practice, Zhejiang Hospital, Hangzhou, Zhejiang China

**Keywords:** Six-minute walking test, Reference equation, Exercise testing, Obese people

## Abstract

**Background:**

Studies have shown that the reference equations for the six-minute walking distance (6MWD), which were mainly derived from healthy, normal-weight people, are not suitable for individuals with obesity. The main purpose of this study was to establish reference equations for the 6MWD in obese Chinese subjects.

**Methods:**

In our study, a total of 214 individuals with obesity performed the six-minute walking tests (6MWTs) according to the American thoracic society (ATS) guidelines, and the longer 6MWD was used for further analysis. The reference equations for the 6MWD were developed using stepwise multiple regression analysis. The newly established equations for the 6MWD were compared to the existing prediction equations.

**Results:**

The mean 6MWD for the cohort was 523 ± 56 m. We found that the reliability of two 6MWTs was good. Age and BMI were identified as independent factors, and explained 31% and 27% of the variance in the 6MWD for the male and female participants, respectively. Thus, the reference equations reported in the previous studies did not accurately predict the 6MWD in our subjects.

**Conclusion:**

Our study was the first to describe the 6MWD in obese Chinese subjects and to propose new predictive equations. These established equations can improve the assessment of the health of obese Chinese patients whose exercise capacity is affected by the disease.

**Level of evidence:**

III, Cohort study.

## Introduction

Given the obesity pandemic, clinicians and researchers have begun to explore effective diagnostic tools for assessing functional capacity and cardiovascular health. Relevant experiments, such as walking tests, have been conducted to assess functional capacity in a safe and reasonable way; these tests can serve monitor health [1]. The six-minute walking test (6MWT) reflects the physical activities of daily living and is a simple and inexpensive tool for measuring cardiovascular fitness [2]. The 6MWT is a commonly used clinical assessment of functional capacity, monitoring treatment progress and developing prognoses in patients with cardiopulmonary disease [3–5]. The 6MWT is used in a wide range of conditions, including stroke, Down syndrome, Alzheimer's disease, cerebral palsy, and chronic pain conditions [6–13]. In addition to these, the use of the 6MWT has been extended to a tool for assessing the physical functioning of patients with obesity and monitoring improvements following interventions (physical therapy, exercise, and weight loss) [14–16].

Since 2002, the American Thoracic Society (ATS) has provided and published comprehensive guidelines for the 6MWT, which define its inclusion criteria and provide implementation instructions and interpretation parameters [17]. Although the 6MWT has been widely used, it has major problems. In particular, the 6MWT, as a tool, has been used for the clinical assessment of functional capacity in patients with obese Chinese people, but there are no specific effective experimental data. In the previous studies, although we have established reference equations for the six-minute walking distance (6MWD) of healthy Chinese subjects [18, 19], these equations are not applicable to subjects with BMI > 30 kg/m^2^. Some studies [20] have shown that reference equations for the 6MWD, which were derived mainly from healthy, normal-weight populations, are not suitable for obese subjects and that specific reference equations for the 6MWD in obese subjects should be established. Although 6MWD reference equations derived from obese subjects of other ethnicities have been established [9, 21–23], these equations may not be applicable to obese Chinese people. The lack of 6MWD reference equations for obese Chinese subjects limits the applicability of the 6MWT for obese patients and makes it difficult for clinicians who use the 6MWD.

The main purposes of this study were as follows: (1) to determine standard measurements of anthropometric variables and walking distance for obese Chinese people more than 40 years old according to the measurement standard stipulated by the ATS guidelines; (2) to establish reference equations for the 6MWD in obese Chinese people; and (3) to compare the 6MWD reference equations obtained in this study with the previous equations for subjects in the same age range [21, 24–26].

## Methods

### Subjects

We collected data over a 17-month period from May 2019 to December 2020. Obese subjects (BMI > 30 kg/m^2^) more than 40 years old were recruited from three local communities. Participants were briefed on the purpose of the study prior to recruitment to motivate test completion. We obtained written informed consent from participants before the study. The subjects completed a health questionnaire before participating in the 6MWT, and researchers verified the results of the questionnaires. This study was approved by the Ethics Committee of Wenzhou People’s Hospital.

The exclusion criteria for subjects were as follows:

- age > 75 years;
- self-reported disease symptoms (including heart disease, lung disease, or other blood or neurological diseases);
- baseline heart rate (HR) < 50 bpm or ≥ 100 bpm;
- baseline systolic blood pressure (SBP) ≥ 180 mmHg or diastolic blood pressure (DBP) ≥ 100 mmHg;
- trouble walking;
- needing for a hearing aid.

### Physical examination

The subjects underwent anthropometric measurements, which were performed by skilled experimenters following the procedures described in the anthropometric standardization reference manual. Age was verified by each subject’s identity card. Height was measured to the nearest 0.1 cm with a height gauge; for this measurement, the subjects were instructed to take off their shoes and stand with their back straight. An electronic scale was used to measure body weight (kg) to the nearest 0.1 kg, and the BMI algorithm = weight/height^2^ (in units of kg/m^2^) was used to calculate the BMI of each subject.

### Six-minute walking test

Both 6MWTs were carried out in an enclosed space: a 30-m-long corridor with a straight, flat surface. The space was marked by a distance marker every 3 m, and orange traffic cones were placed at the turning points of the walk. The starting line represented both the starting point and the end of the 60 m, with colorful tape on the floor serving as a reminder. The room temperature for the test needed to be held between 20 and 25 ℃, so the test was only conducted between 9:00 AM and 1:00 PM. Participants had to follow strict rules such as no strenuous exercise or eating before the start of the test; they had to rest in a chair near the starting point for 10 min or more and then undergo measurements of resting blood pressure and heart rate prior to the test. Before the start of the test, the subjects were told that the purpose of the test was to measure how far they could normally walk in 6 min. If the subjects experienced dizziness, chest pain, difficulty breathing, or leg cramps during the test, they were allowed to stop and rest, but once they were well again, they were encouraged to continue walking quickly. To ensure experimental rigor, an experimenter participated in two 6MWTs to monitor the data. The experimenter also provided standard encouragement to the participants every 60 s (e.g., “you are doing well; you have five minutes left”, “good job; there are four minutes left”) [17]. The distance the subject had walked at 6 min was recorded as the 6MWD. At the beginning of the experiment and after the completion of the 6MWT, each subject was presented with the Borg dyspnea scale [27]. On a scale ranging from “0 = none” to “10 = very, very severe,” the subjects used the scale to assess their current degree of shortness of breath. Two hours later, the subject's second test was completed by the same experimenter.

### Statistical analysis

The Kolmogorov–Smirnov test was used to check whether the predicted data were normally distributed. The measured data were expressed as the mean ± standard deviation (SD) or as numbers and percentages, as appropriate. Descriptive analysis was used to analyze the characteristics of the subjects. Independent Student’s *t* test was used to illustrate the relationship between the 6MWD and the categorical variables. In this study, we used paired-samples Student’s *t* test to compare the observed 6MWD in our subjects and the predicted 6MWD based on the previously published reference equations derived from other studies [21, 24–26]. The repeatability of the two 6MWDs was calculated according to Bland–Altman analysis and calculation of the intraclass correlation coefficient (ICC) [28]. First, the correlations between the 6MWD and subject characteristics (i.e., age, height, weight, and BMI) were individually evaluated by Spearman’s correlation analysis. Second, the reference equations of the 6MWD were established by forwards stepwise multivariate regression analysis. In each procedure, the most significant categorical variable was added to the model, and then, the same procedure was followed until no additional statistically significant variables remained. A *p* value > 0.05 was used to detect whether the variable was entered or deleted. Data were analyzed using SPSS statistical software for Windows (version 20.0; SPSS, Inc., Chicago, IL). A *p* value < 0.05 was considered significant in all analyses.

## Results

### Demographic characteristics and 6MWT results

Two hundred ninety-five obese subjects were recruited for this study. Eight-one subjects were excluded from the study (foot sprain: *n* = 2; cardiac disease: *n* = 26; abnormal basal heart rate: *n* = 10; unstable hypertension: *n* = 20; pulmonary disease: *n* = 6; and cerebral disease: *n* = 17). Finally, 214 obese subjects (102 men and 112 women) completed the 6MWTs, with no subjects prematurely requiring rest during the test or terminating the test. Table 1 shows the characteristics of these subjects and the 6MWT results. Male participants were significantly taller and heavier than females in our study, and there was a corresponding difference in BMI between the sexes. The mean 6MWD for all the subjects was 523 ± 56 m. The mean distance was 540 ± 57 m for male participants and 508 ± 51 m for female participants; this difference was significant (*p* < 0.001). Age- and sex-stratified values of the 6MWD are summarized in Table 2. The mean 6MWD of the first and second test sessions were 508 ± 53 m and 517 ± 55 m, respectively. The reliability of two 6MWTs was good (ICC = 0.89). We also detected a difference in heart rate between the sexes. By the end of the test, subjects' heart rates had reached our predicted maximum of approximately 65%.

**Table 1:** Characteristics and 6MWT results of the study subjects

Characteristic	Males (*n* = 102)	Females (*n* = 112)	*p* value*	Total (*n* = 214)
Age, years	56.9 ± 10.23	57.9 ± 10.22	NS	57.5 ± 10.21
Height, cm	166.9 ± 6.29	154.4 ± 5.40	< 0.001	160.4 ± 8.57
Weight, kg	87.4 ± 7.26	76.0 ± 6.87	< 0.001	81.4 ± 9.04
BMI, kg/m^2^	31.3 ± 1.28	31.9 ± 1.75	< 0.05	31.6 ± 1.57
HR1, bpm	74.3 ± 8.99	71.8 ± 8.53	< 0.05	73.0 ± 8.82
HR2, bpm	106.4 ± 11.87	104.4 ± 11.53	NS	105.4 ± 11.71
Borg	2.1 ± 0.70	2.2 ± 0.37	NS	2.1 ± 0.57
6MWD, m	539.6 ± 57.18	508.2 ± 51.36	< 0.001	523.2 ± 56.33

Values are expressed as the mean ± SD

*p value between males and females

**Table 2:** Age and gender stratified norms of the 6MWD

Age, years (*n*)	Males (*n* = 102)	Females (*n* = 112)	*p* value*	Total (*n* = 214)
40–49 (*n* = 62)	557.5 ± 57.31	528.1 ± 55.32	< 0.05	543.3 ± 57.83
50–59 (*n* = 63)	549.4 ± 54.90	520.9 ± 45.07	< 0.05	534.9 ± 51.76
60–69 (*n* = 56)	538.6 ± 49.54	510.0 ± 44.71	< 0.05	523.3 ± 48.74
70–75 (*n* = 33)	474.5 ± 26.16	455.0 ± 22.15	< 0.05	462.7 ± 25.32

Values are expressed as the mean (range)

*6MWD* six-minute walking distance

*p value between males and females

### Associations with the 6MWD

Table 3 shows the summary of the relationships between the 6MWD and the variables measured for male and female participants. According to the univariate linear regression analysis, the variables (age, height, and BMI; presented in Table 3) were obviously correlated with the 6MWD. These variables (age, height, and BMI) were included in the stepwise multivariate regression analysis. Age and BMI were identified as independent factors that influenced the 6MWD and explained 31% and 27% of the variance in distance for the male and female participants, respectively (Table 4).

**Table 3:** Pearson correlations between the variables and the 6MWD

Variable	Males (*n* = 102)		Females (*n* = 112)	
	*r* value	*p* value	*r* value	*p* value
Age	− 0.397	< 0.001	− 0.442	< 0.001
Height	0.167	< 0.05	0.164	< 0.05
Weight	− 0.030	NS	− 0.030	NS
BMI	− 0.359	< 0.001	− 0.263	< 0.05

*6MWD* six-minute walking distance, *r value* Pearson’s correlation coefficient, *BMI* body mass index

**Table 4:** Results of stepwise multiple linear regression analysis of the independent variables that explained the 6MWD

	Males			Females		
	B	SE	*p* value	B	SE	*p* value
Constant	1261.349	122.118	< 0.001	923.596	81.351	< 0.001
Age	− 2.509	0.465	< 0.001	− 2.342	0.409	< 0.001
BMI	− 18.484	3.701	< 0.001	− 8.783	2.382	< 0.001
*R*^2^	0.327			0.285		
Change in *R*^2^	0.314			0.272		

*B* unstandardized coefficients

The reference equations for the 6MWD were as follows:

$$\begin{gathered} {\text{Male:}}\;{\text{6MWD}}\left( {\text{m}} \right) = {1261}{\text{.349}} - \left[ {{\text{age}}\left( {{\text{yr}}} \right) \times {2}{\text{.509}}} \right] - \left[ {{\text{BMI}}\left( {{\text{kg/m}}^{{2}} } \right) \times {18}{\text{.484}}} \right];r^{2} = 0.{314} \hfill \\ {\text{Female:}}\;{\text{6MWD}}\left( {\text{m}} \right) = {923}{\text{.596}} - \left[ {{\text{age}}\left( {{\text{yr}}} \right) \times {2}{\text{.342}}} \right] - \left[ {{\text{BMI}}\left( {{\text{kg/m}}^{{2}} } \right) \times {8}{\text{.783}}} \right];r^{2} = 0.2{72} \hfill \\ \end{gathered}$$

### Comparison with published reference equations

Table 5 shows a comparison between the measured 6MWD in our subjects and the 6MWD predicted by the previous reference equations [21, 24–26] for the same age ranges. The previously published reference equations did not accurately predict the 6MWD in our subjects, and there was a significant difference between the observed and predicted 6MWD within the same age range (*p* < 0.05). The 6MWD in our subjects was overestimated by reference equations derived from previous studies.

**Table 5:** Measured 6MWD and predicted 6MWD for the same age range based on the equations reported in previous studies

Study	Measured (m)	Predicted (m)	Measured–predicted (m)
Ben Saad et al. [24]	523.2 ± 56.33	559.9 ± 101.93	− 72.7 ± 90.03*
Iwama et al. [25]	523.2 ± 56.33	545.7 ± 36.88	− 22.5 ± 51.78*
Capodaglio et al. [21]	539.7 ± 54.77	601.2 ± 30.20	− 61.5 ± 51.75*
Camarri et al. [26]	508.1 ± 49.37	615.8 ± 42.54	− 143.7 ± 48.52*

*6MWD* six-minute walking distance

*p < 0.05 according to Student’s *t* test

## Discussion

To the best of our knowledge, this was the first study to describe 6MWD in obese Chinese subjects more than 40 years old. As shown in our study, there was a significant sex difference in the walking distances of men and women. Men tend to walk longer distances than women over the same span of time, probably due to their higher muscle mass and greater athletic ability.

**Fig. 1:**
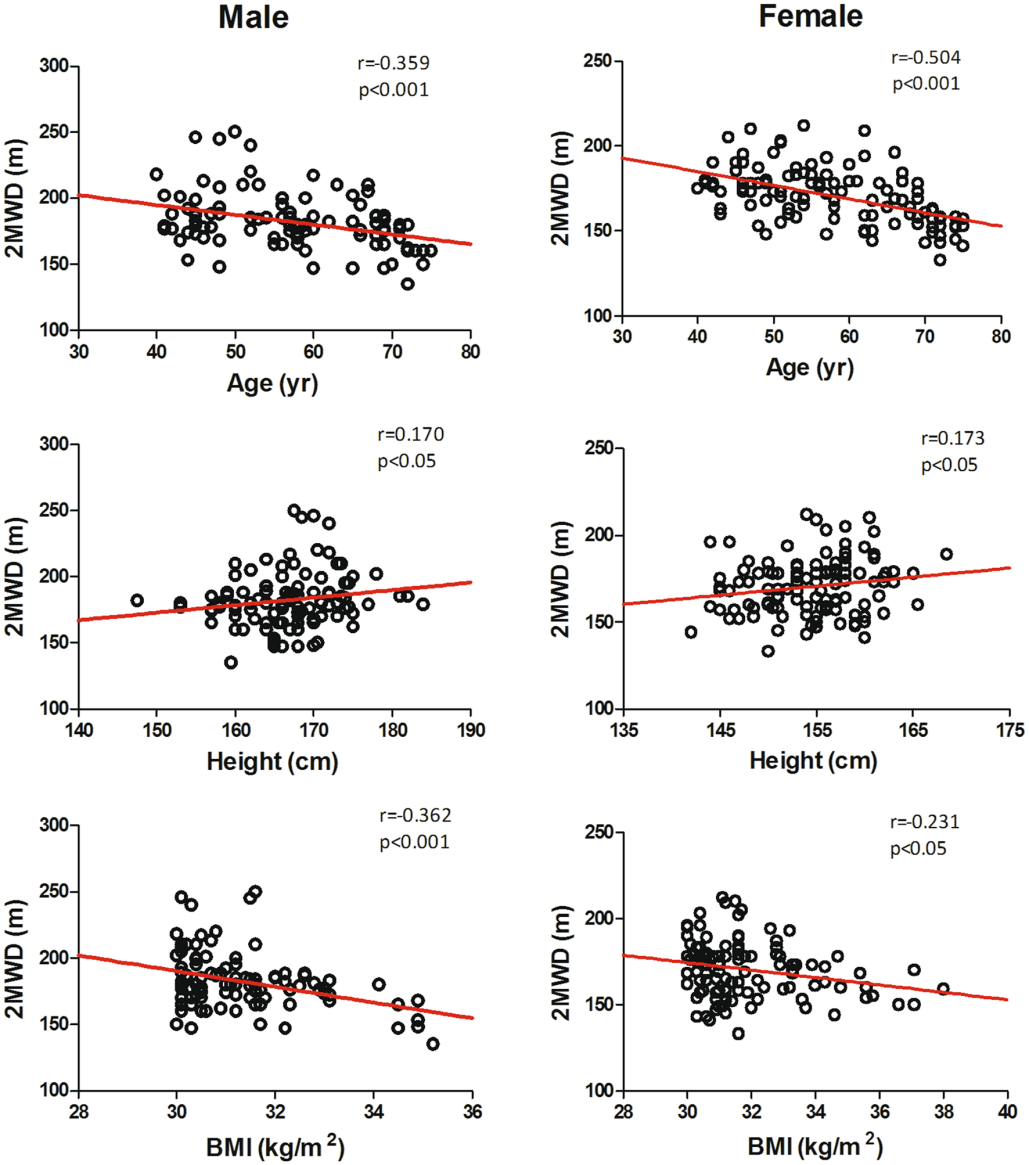
The relationship between the 6MWD and age, height, and BMI in men and women

There was a correlation between the 6MWD and independent variables in both men and women (Fig. 1). Age and BMI were negatively correlated with the 6MWD in our study. This is probably related to the loss of muscle mass with age and the decrease in oxygen intake. Obese subjects experience certain complications with an increase in BMI, which usually manifest as activity disorders caused by heart and breathing limitations [29, 30], and weakened skeletal muscle strength is also one of the main causes of disability [31, 32]. Some possible reasons include skin friction caused by fat deposition in the thighs, increased plantar pressure, and physical discomfort caused by exercise for people with a BMI that is higher than normal [9, 33–35]. In addition, obesity is often associated with chronic pain, walking distance might also be limited by pain from overloaded joints and relatively weak muscles [35]. We found that height and walking distance were positively correlated in our study. One explanation is that, in general, taller individuals with larger strides cover more ground over the same amount of time than shorter individuals. However, the target of our study was obese subjects, and the BMI was more meaningful than height for predicting the 6MWD, so height was not included in the final regression equation. Additionally, we observed that the resting heart rate of male subjects was lower than that of female subjects. Previous studies have shown sex differences in heart rate, and the resting heart rate of men is lower than that of women [36]. This may be because men and women have different abilities to regulate the baroreflex heart rate, and oestrogen influences the ability to regulate the baroreflex heart rate in people [37].

In this study, categorical variables (age, height, and BMI) were included in the stepwise multiple regression analysis. Age and height were identified as independent factors that influenced the 6MWD, and they explained 31% and 27% of the variance in walking distance for the male and female participants, respectively.

After analysis of the data, such as the theory proposed by Hulens et al. [15], we propose some factors that correlate with walking distance in 6 min that would certainly benefit the predictive value of the reference equations. Other factors (heart rate, blood pressure, muscle strength, chronic pain [38], and lifestyle factors) also play an important role in predicting the 6MWD, but they are impractical for clinical use.

**Fig. 2:**
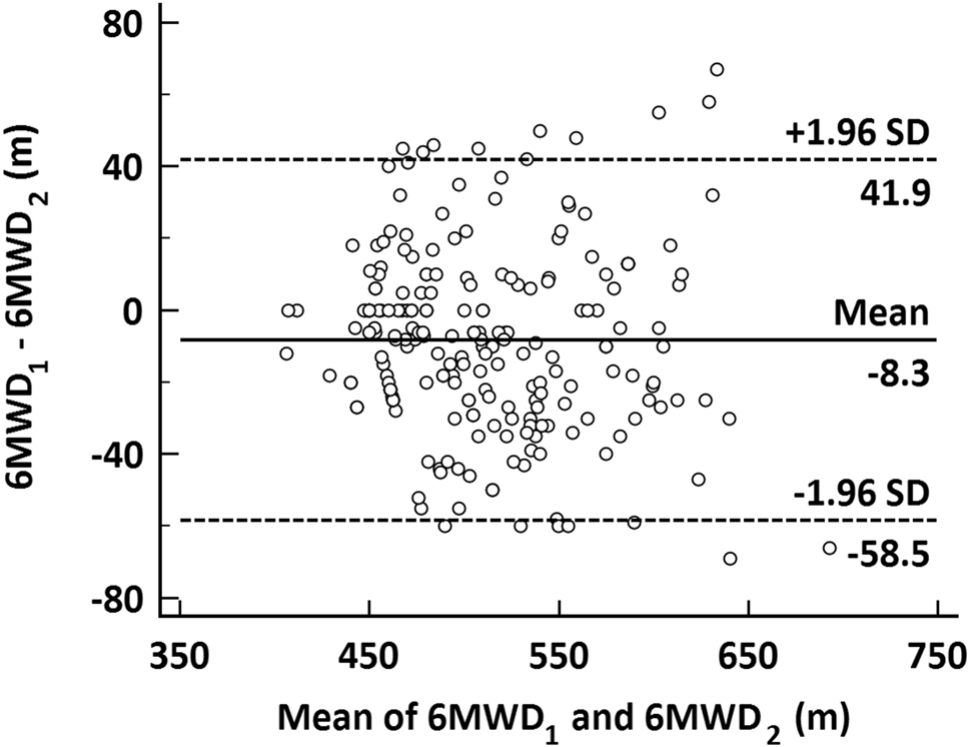
Bland–Altman plot participant performance in the first and second 6MWTs

By measuring the walking distance performance on two occasions, we found that the mean 6MWD during the second test was longer than the mean 6MWD during the first test. This finding is consistent with the previous findings for the 6MWT [5, 39]. This increase in distance is probably related to overcoming negative emotions, improving coordination, and finding an appropriate stride distance. Although the performance of walking distance in the second 6MWT was higher than that of the first test in our study, the reliability of the two 6MWTs was still good (ICC = 0.89). Previous studies have also shown that the two 6MWTs are reliable [39], which is consistent with our results. The Bland–Altman plot shows the mean difference between the first and second 6MWD (Fig. 2). Seventeen participants had error values outside the 95% confidence interval (CI), and seven participants showed an increase in the second 6MWD, which might be due to familiarity with the 6MWT. Ten participants showed shorter walking distances in the second 6MWT, which might be due to greater fatigue during the second test due to better performance in the first 6MWT.

These reference equations from previous studies (Ben et al. [24], Iwama et al. [25], Capodaglio et al. [21], and Camarri et al. [26]) did not accurately estimate the distance walked during the 6MWT for obese subjects. Judging from our data, the walking distance of obese individuals was significantly different from the distance estimated by the reference equations mentioned above. These differences could be due to the test protocol, anthropometric factors, ethnic background, and demographic differences among the participants. In the studies by Ben et al. [24], Iwama et al. [25], and Camarri et al. [26], healthy subjects were primarily recruited, and obese subjects accounted for only a small percentage of the study sample; thus, the walking distance in these studies was higher than the walking distance in our sample. In contrast, Capodaglio et al. [21] also recruited obese participants, but our results are not consistent with their findings. The difference may be due to the higher average age (20 years older) and the lower capacity of the subjects in our study. In a sense, age is a proxy of disability, and obviously affects the performance of subjects during the 6MWT. In our sample, 42% of subjects were over 60 years old, and 15% over 70 years old, whereas in the study by Capodaglio et al.’s study [21], participant ages ranged from 20 to 60 years. Even if age was considered as an explanatory variable [21], the equation was verified in samples younger than 60 years old, thus reducing its predictive validity for elderly subjects. In addition to the daily physical activity of the participants, their mood and psychological factors may influence the 6MWD [40].

Our study also had some limitations. First, although the sample size of our study was relatively large, the sample was one of convenience, and relatively few subjects were over the age of 70 in the study. Second, we did not recruit obese subjects under 40 years of age. A large multicentre study is needed to address these limitations.

In summary, our study was the first to describe the 6MWD in obese Chinese subjects and to propose predictive equations. These newly established equations improve the assessment of health in obese Chinese obese patients with diseases that affect their exercise capacity.

## What is already known on this subject?

The 6MWT is a commonly used clinical assessment of functional capacity, and is used to monitor treatment progression and establish prognoses in patients with cardiopulmonary disease. Although the reference equations of the 6MWD for healthy Chinese subjects have previously been established, some studies have shown that the reference equations for the walking distance derived mainly from healthy, normal-weight people are not suitable for obese subjects. However, there is a lack of standard reference equations for the 6MWD in obese Chinese subjects.

## What this study adds?

The study was the first to describe the 6MWD in obese Chinese subjects and to propose predictive equations. These established equations can contribute to improving the assessment of obese Chinese patients whose exercise capacity is affected by the disease.

## Abbreviations

6MWT: Six-minute walking test
6MWD: Six-minute walking distance
BMI: Body mass index
HR: Heart rate
SpO_2_: Oxygen saturation
SBP: Systolic blood pressure
DBP: Diastolic blood pressure
ICC: Intraclass correlation
SD: Standard deviation
SPSS: Statistical Package for the Social Sciences
